# Patterning ECM microstructure to investigate 3D cellular dynamics under multiplexed mechanochemical guidance

**DOI:** 10.12688/f1000research.125171.1

**Published:** 2022-09-20

**Authors:** Pedram Esfahani, Bo Sun

**Affiliations:** 1 Physics, Oregon State University, Corvallis, OR, 97330, USA

**Keywords:** extracellular matrix, tissue engineering, contact guidance, chemotaxis, 3D culture

## Abstract

**Background:** Biochemical and biophysical factors jointly regulate the cellular dynamics in many physiological processes. It is therefore imperative to include multiplexed microenvironment cues when employing {in vitro} cell-based assays to model physiological processes.

**Methods:** To meet this need, we have developed a modular platform of 3D cell culture, Modular Control of Microenvironment for Cell Migration and Culture Assay (MC33A), that incorporates directed chemical and mechanical cues in the forms of chemotaxis and contact guidance, respectively. Taking advantage of the functionalities of MC33A, we study the migration and morphology of breast cancer cells in 3D engineered extracellular matrix (ECM) following a serum gradient for chemotaxis.

**Results:** We show that when chemotaxis is facilitated by contact guidance in the same direction as the serum gradient, cells demonstrate dimensional-reduction in their motility and highly elongated ellipsoidal shape. When the direction of ECM alignment diverges from the direction of serum gradient, chemotactic motion is significantly suppressed, and cells are generally more protrusive and rounded in their morphology.

**Conclusions:** These examples demonstrate MC33A as a powerful tool for engineering complex microenvironments of cells that will advance the state-of-the-art of cell-based analysis in drug development, regenerative medicine, and other research areas in bioengineering.

## Introduction

Many physiological processes involve directed mechanochemical cues that regulate the motility, polarization, and morphogenesis of cells such as during wound healing, ^1^ immune response, ^2^ and cancer metastasis. ^3^ It is, therefore, crucial to incorporate these extracellular signals when employing *in vitro* cell-based assays in applications such as drug screening and tissue regeneration. ^4^^,^^5^ Here we present a modular platform to study cellular dynamics in a 3D extracellular matrix (ECM) where directed chemical and mechanical cues, in the forms of chemotaxis and contact guidance, are fully controlled in the microenvironment of cells.

As one of the most common types of directed chemical cues, ^6^^,^^7^ chemotaxis is the process where a cell follows the gradient of chemoattractants to coordinate with other cells in the functions of multicellular organisms. ^8^^,^^9^ Contact guidance, on the other hand, utilizes the substrate or tissue topography to direct the cells through mechanosensing pathways. ^10^^–^^12^ Contact guidance strongly modulates the morphology and motility of cells, as have been observed in many cell types. ^13^^–^^17^

Although the effects of chemotaxis and contact guidance have been well studied separately, accurate representation of physiological conditions requires simultaneous presence and control of mechanochemical cues. One salient example is cancer metastasis, where the gradient of various growth factors drives chemotaxis, which facilitates the cancer cell dissemination. ^18^ Concurrently, cells move through vast tissue space filled by fibrous ECM and the alignment of ECM fibers generates contact guidance. ^19^ As a result ECM microstructure significantly correlates with tumor prognosis. ^16^^,^^20^

While it is desirable to program complex mechanochemical cues in cell-based assays, a reliable and user-friendly method has not been available to the broader community. To incorporate both biochemical and biophysical factors in the microenvironment of the cells, we have developed techniques to pattern the microstructure of 3D extracellular matrices. We have packaged these techniques into a portable modular platform: Modular Control of Microenvironment for Cell Migration and Culture Assay (MC ^3^A). MC ^3^A simultaneously controls mechanochemical factors for 3D cultured cells, with a form factor that is compatible with standard microscopy for live or fixed cell imaging.

To demonstrate the functions of MC ^3^A, we study the migration and morphology of breast cancer cells in 3D ECM. We engineer the extracellular microenvironment to simultaneously establish a chemotactic serum gradient, and contact guidance that is either in converging or diverging directions of chemotaxis. These examples will show MC ^3^A as a valuable tool to study cellular dynamics and functions in realistic tissue environments.

## Methods

We used OEM, and 3D printed parts to build the prototype of MC ^3^A and followed the manufacturer’s instructions to prepare the 3D ECM and cell culture. We analyzed the images using ImageJ (RRID:SCR_003070) plugins and homemade scripts compiled with MathWorks MATLAB (RRID:SCR_001622). As an open source alternative to MATLAB, GNU Octave (RRID:SCR_014398) can be employed to render the same results. Microscopy images were directly analyzed for ECM geometry and cell segmentation without any modifications. Microscopy images shown in Figure 3 and Figure 5 are contrast enhanced to facilitate visual inspection.

### Cell culture

We culture RFP-labeled MDA-MB-231 human breast cancer cell line (GenTarget, San Diego, CA), following the standard protocol, in a high glucose Dulbecco’s modified Eagle’s medium supplemented with 10 *%* fetal bovine serum (FBS; Avantor Seradigm, Radnor, PA) and 0.1 *%* Penicillin-Streptomycin (Gibco Thermofisher, Waltham, MA), and maintained it at 37°C and 5 *%* CO _2_ incubator.

Prior to conducting experiments, for precisely 12 hours, we culture the cells in a serum-free medium containing Dulbecco’s modified Eagle’s medium supplemented with 0.1 *%* Penicillin-Streptomycin Gibco Thermofisher, Waltham, MA, and maintained at 37°C and 5 *%* CO _2_ incubator.

To embed the cells in 3D collagen matrices, we suspend the cells at low density in neutralized collagen solutions. Highly concentrated rat-tail FITC-labeled collagen is diluted with 10× L15 medium (Dulbecco), DDI water, NaHCO _3_ 7.5 *%*, and neutralized with sodium hydroxide (NaOH, 1M) to a final concentration of 1.5 mg/mL with a pH of 7.4.

### Sample preparation on the culture inserts

The culture assays are first corona treated for 10 min, then immersed with Sulfo-SANPAH under UV light (320-350 nm) for 2 hours to functionalize binding surfaces. Then we wash the assays with 1mL of PBS 1× and DDI water. After drying the samples, we transfer the 3D cell suspension solution to the assay using 100 um pipet tips through the inner channel port (center port).

Then, using the rotary stage, we place the treated blade at the center of the assay and rotate it for a given time with a defined RPM (128RPM for 4 min as in the main text). Following this process, we polymerize the solution for 21 minutes at room temperature and then for 25 min in a 5 *%* CO _2_ incubator at 37°C. Then we add 1 mL of a serum-free growth medium to each reservoir of the assays and keep it in the incubator for 6 hours (serum starvation period). Rotation with zero RPM results in radially aligned ECM.

After transferring the assays to the microscope, we replace the center reservoirs growth medium with a 20 *%* serum-rich and the outer one with a serum-free L15 medium to generate a serum gradient across the chemotaxis channel. After 6 hours, the sample is ready for imaging.

### FITC-labeled type I collagen ECM

850 μL of 10 mg/ml Collagen Type I, Rat Tail (purchased from ibidi GmbH, Grafelfing, Germany) mixed with 150 μL FITC conjugated water-soluble Collagen (Type I) (obtained from AnaSpec, Fremont, CA). We store the solution at 4°C and in the dark. We gently mix it once a day for about 30 seconds for ten days prior to experiments. The final concentration of the collagen solution is 1.5 mg/mL after adding cell suspension.

### Microscopy

We use a Leica TCS SPE confocal microscope equipped with a stage-top incubator. We capture the pictures at a rate of 1 frame per hour over 18 hours using a 10× air objective. The raw images are grayscale with a resolution of 1024×1024 pixel ^2^ (1.1×1.1 mm ^2^) along the x-y plane and with two slices at a step of 100 μm along the z-direction. We simultaneously use the multichannel capability to image collagen fibers and MDA-MB-231 cells.

### Image processing

Images are analyzed using custom scripts compiled using MathWorks MATLAB and Python 3. First, cell images are binarized to obtain cell objects. Connected components are found and manually screened to represent single cells. We calculate cell centroids and morphology from binarized images.

To calculate the local collagen orientation and coherence, we use the ImageJ plugin OrientationJ. ^21^ This software package computes the orientational order of an image based on its gradient matrix. With a sliding window, one can obtain the field of local principal direction and coherence *c.* The principle direction indicates the direction along which the image intensity vary minimally, while the coherence *c* measures the level of alignment in the local structure. When all fibers are in parallel, *c* reaches a maximum value of 1. When the fibers are randomly aligned *c* approaches its minimal value of 0. Note that the imaging noise generally suppresses the calculated coherence. And the intrinsic disorder of biopolymer networks forbids perfect alignment. Therefore the theoretical upper bound of 1 can not be reached. By comparing calculated coherence with visual inspection and cellular responses, we consider collagen fibers to be well aligned when *c* > 0.2.

## Results

**Figure 1.**
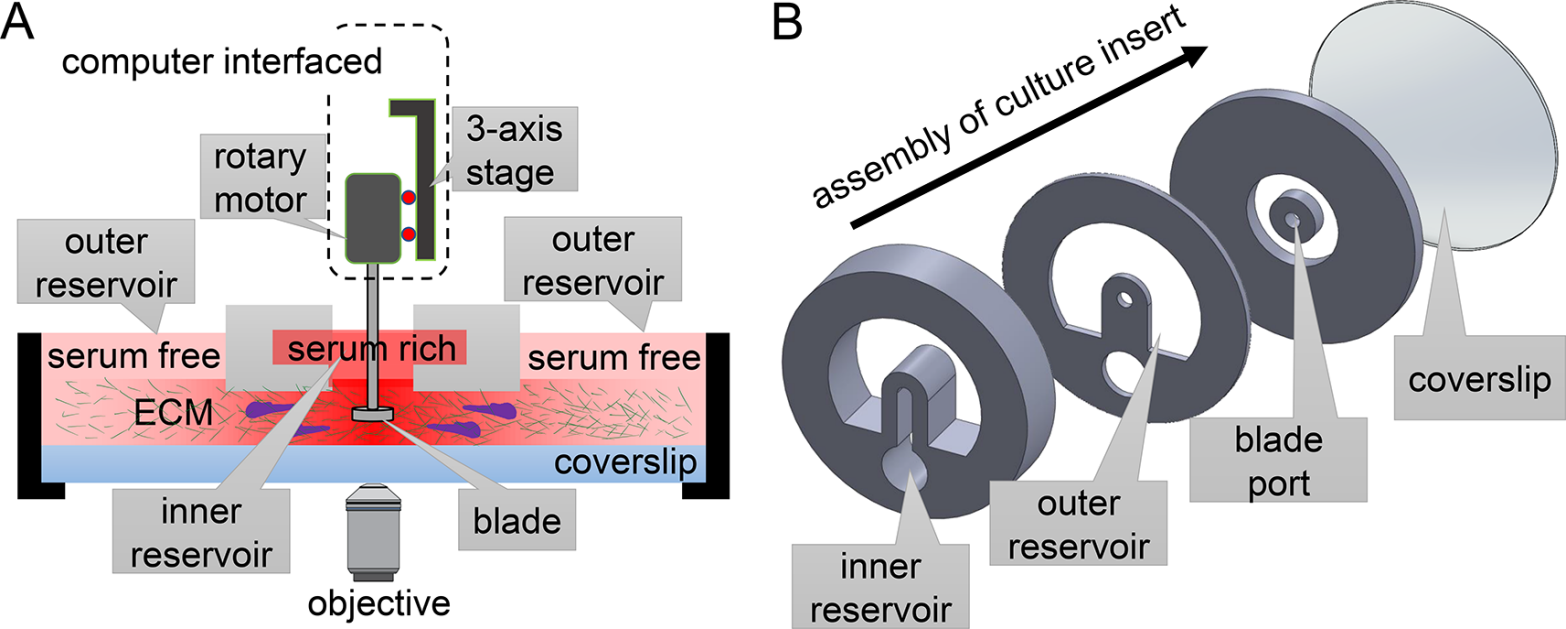
Schematic design of Modular Control of Microenvironment for Cell Migration and Culture Assay (MC ^3^A). (A) The rotary stage of MC ^3^A harbors multi-axis translation and rotation motors to manipulate a blade. These motors are interfaced with a computer that instructs the self-assembly of extracellular matrix (ECM) proteins into fibrous architectures. (B) The exploded view of a culture insert, which contains ECM for 3D cell culture and motility assay. The geometry of the culture insert can be altered to produce various types of microenvironments for cells, therefore offering an expansion of functions. The design presented here allows simultaneous chemical gradient and mechanical cues from ECM fibers with programmable alignment.

MC ^3^A consists of a rotary stage that is controlled through computer interface, and a disposable culture insert ( Figure 1, see also extended data section S1-S2 ^32^). The rotary stage incorporates translation motors to move a spinning head. The spinning head consists of a blade coupled with a rotary motor. When making samples, these motors are manually controlled or follow pre-programmed protocols to dip the blade through the blade port of culture inserts. After facilitating the ECM self-assembly, user uses the z-motor to lift up the blade and the culture insert can be manipulated or imaged in ways similar to a standard tissue culture petri dish.

To produce desirable ECM microstructure, MC ^3^A takes advantage of a blade with experimentally optimized tip shape to guide the gelation of ECM polymer solution. Additionally, spinning of the blade creates a flow in the ECM solution. The flow is both driven by the rotational motion of the blade, as well as constriction walls built inside cell culture insert. After setting the initial flow, the blade exits from the solution and the polymer solution solidifies into a biopolymer network whose microstructure is templated by the initial flow. ^17^^,^^22^ The shape of the blade, its rotation protocol, and the geometry of constriction walls of the culture insert work synergistically to make tissue mimicking biopolymer networks.

Above the engineered ECM microstructure, we place a pair of open channel reservoirs to deliver soluble factors. The reservoirs can be filled with the same or different chemicals to control the biochemical microenvironment of the cells. As an example, we would fill the inner reservoir with growth medium supplemented with 20% volume concentration of serum while the outer reservoir with growth medium without serum. The passive diffusion between the reservoir sets up a serum gradient, which drives chemotactic motion of MDA-MB-231 breast cancer cells.

**Figure 2.**
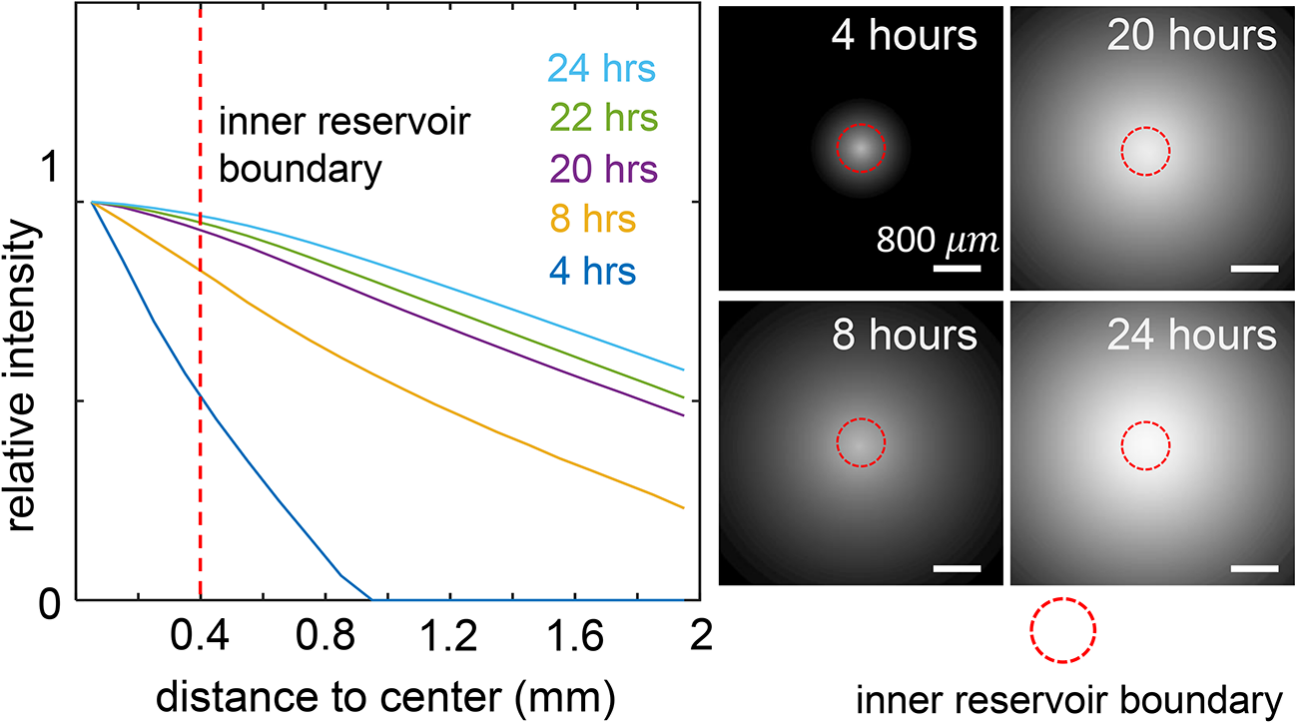
The spatial-temporal profile of diffusive biochemical factors in the culture insert of Modular Control of Microenvironment for Cell Migration and Culture Assay (MC ^3^A) simulated by rhodamine B solution. Left: the relative fluorescence intensity of rhodamine B at varying distances to the center of the inner reservoir, and at different time points. Right: raw fluorescent images at different time points. The fluorescence intensity is symmetric around the center of the inner reservoir. See also *Underlying data*. ^29^

To characterize the microenvironment of cells created by MC ^3^A, we first examined the profile of diffusive factors. To this end, we constructed the ECM with 2 mg/mL type-I collagen matrices (see Methods). We then filled the inner reservoir with rhodamine B in Phosphate-buffered saline (PBS) solution, and filled the outer reservoir with pure PBS. We measured the fluorescence intensity of rhodamine B, which provided a proxy of a diffusive factor’s concentration profile. As shown in Figure 2, ^29^^,^^30^ the fluorescence intensity uniformly expands from the inner reservoir in the radial direction towards the outer reservoir. Eight hours after filling the reservoirs, an approximate linear gradient has established along the radial direction in the ECM outside the inner reservoir. The intensity profile continues to stabilize. After 12 hours of passive diffusion, the intensity gradient in the radial direction reaches a steady state that can last for more than 12 hours. In the current design, the inner and outer reservoirs each have a capacity of 1.5 mL. Increasing the dimensions of the reservoirs, such as by raising the height of the retaining walls, will further elongate the duration of stable chemical gradient.

To characterize the mechanical microenvironment of cells created by MC ^3^A, we employed FITC-labeled type-I collagen so the ECM microstructure can be accessed through fluorescent confocal imaging (see Methods). As representative examples, we examined two distinct configurations produced by executing two simple rotational protocols of MC ^3^A.

**Figure 3.**
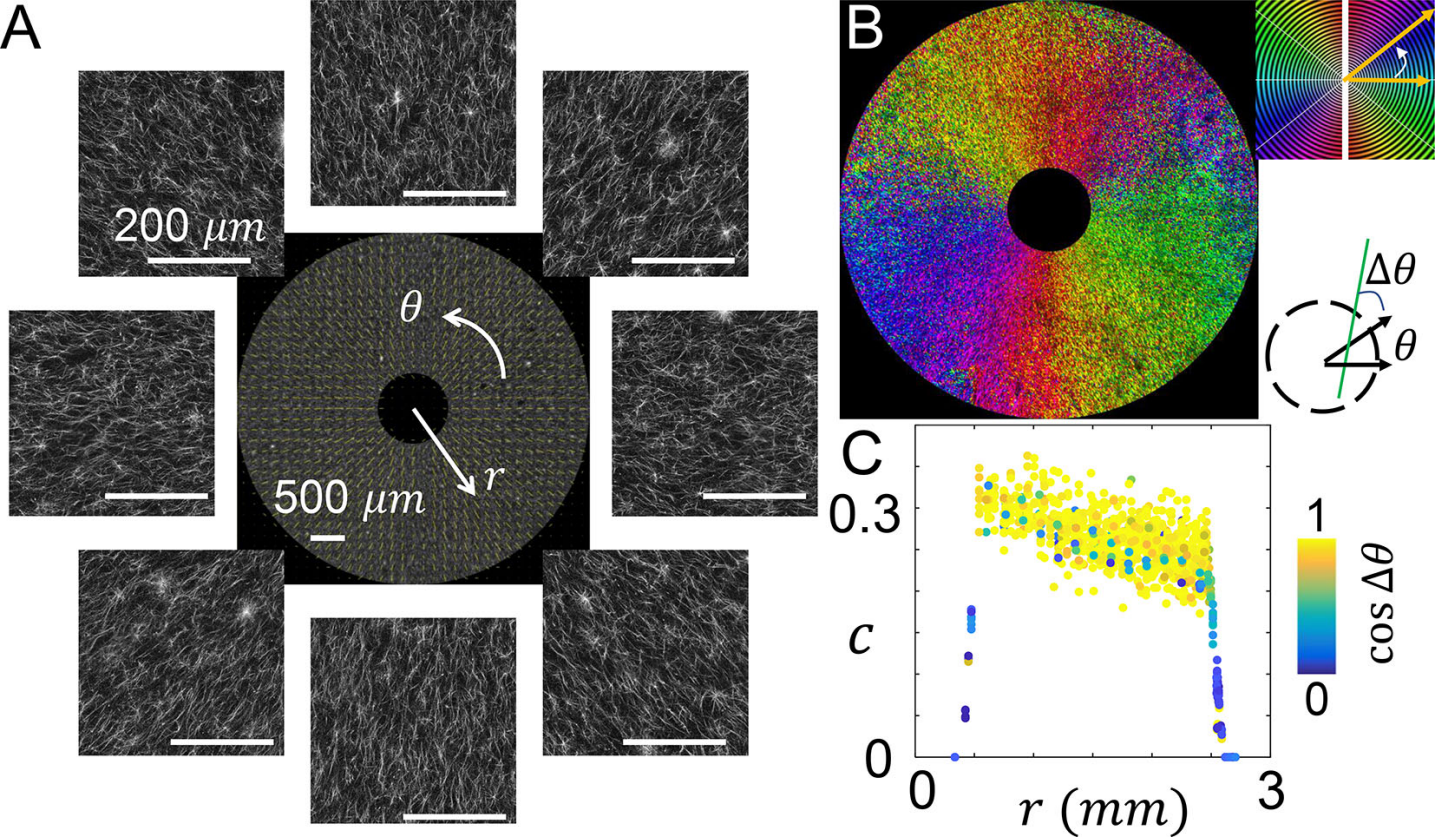
The microstructure of radially aligned extracellular matrix. (A) Confocal images showing the fluorescently labeled collagen fibers. The images are further processed to calculate the principal direction and level of alignment (quantified as coherence) of collagen fibers. Center of the image is at the center of the inner reservoir, similar to Figure 2. (B) A spatial map showing the principal direction of collagen fibers. (C) The coherence *c* at varying distances from the center of the inner reservoir. The data points are color coded by the cosine angle Δ *θ* between local fiber direction (principal direction) and radial direction. See also *Underlying data*. ^30^

When the blade is held still before lifting up from the solidifying collagen solution, the blade combines with the restriction walls in the culture insert to form a boundary condition that facilitates the nucleation of collagen fibers along the radial direction. Confocal images show the expected ECM microstructure ( Figure 3A). To further quantify the local ECM geometry, we calculated the principal direction ( Figure 3B-C) and coherence *c* ( Figure 3C) of collagen fiber alignment as reported previously ^17^^,^^21^^,^^22^ (see Methods). Figure 3B shows the spatial distribution of principal fiber direction. Despite of the fluctuations expected from the disordered nature of biopolymer networks, collagen fibers evidently show alignment in the radial direction.

In addition to the principal direction, coherence *c* measures the level of alignment in the local structure. When all fibers are in parallel, *c* reaches a maximum value of 1. When the fibers are randomly aligned *c* approaches its minimal value of zero. Note that the imaging noise generally suppresses the calculated coherence. And the intrinsic disorder of biopolymer networks forbids perfect alignment. Therefore the theoretical upper bound of one cannot be reached. By comparing calculated coherence with visual inspection and cellular responses, we consider collagen fibers to be well aligned when *c* > 0.2. ^22^

As shown in Figure 3C, the value of coherence starts from around 0.30 near the inner reservoir, and gradually decreases to approximately 0.25 at 2 mm from the device center. The change is well within the range of fluctuations resulted from ECM structural disorder. Therefore the ECM within the culture insert of MC ^3^A demonstrates relatively uniform microstructure.

**Figure 4.**
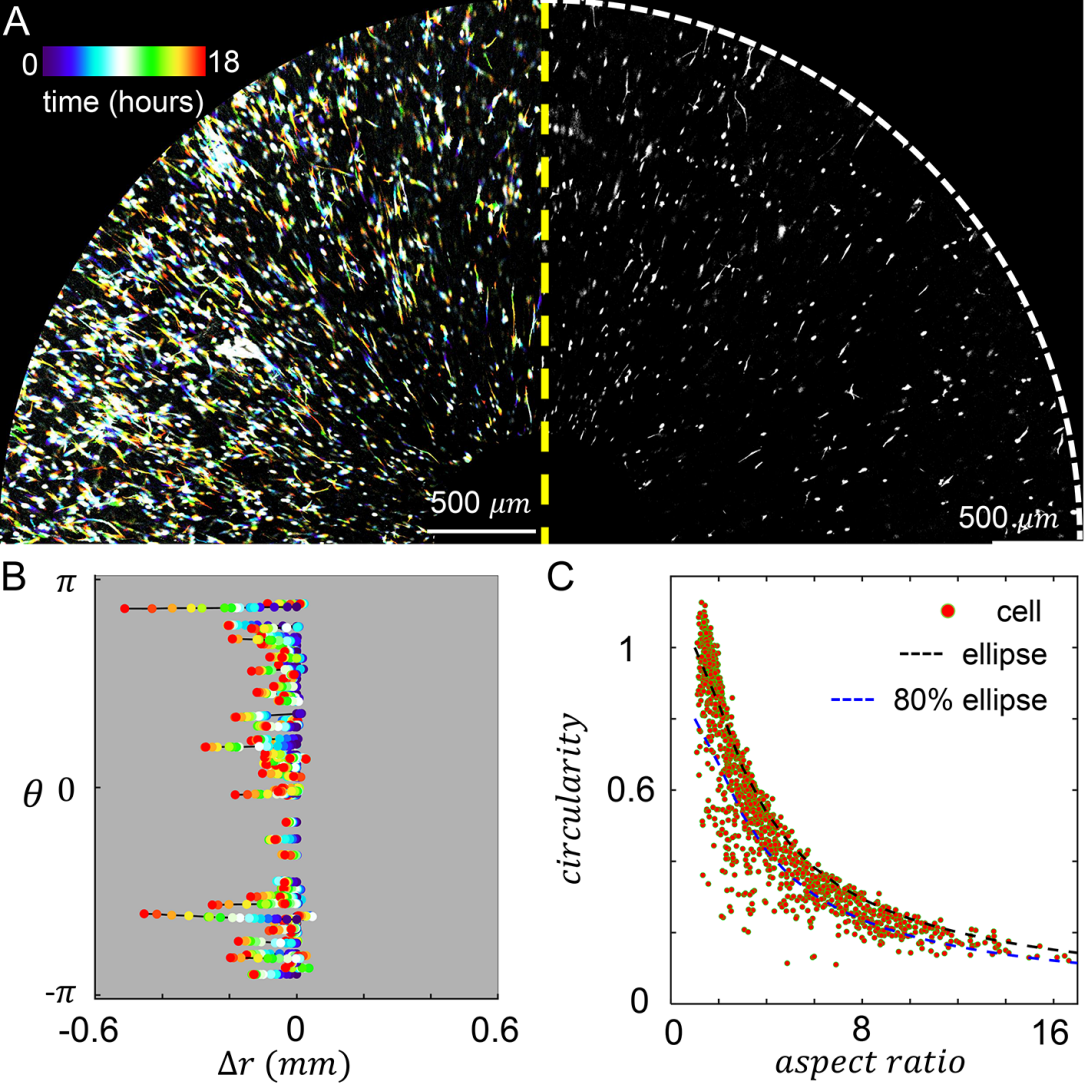
3D Cell motility is modulated by the chemoattractant gradient, which is in parallel to the extracellular matrix (ECM) fiber alignment. (A) Left: temporal projected recording of MDA-MB-231 embedded in the culture insert of Modular Control of Microenvironment for Cell Migration and Culture Assay (MC ^3^A) for 18 hours. Right: a snapshot at five hours. (B) Cell trajectories in the radial (Δ *r*) and azimuthal ( *θ*) coordinates. (C) A scattered plot showing cell aspect ratio and circularity. The black dashed line indicates the circularity of an ellipse at a given aspect ratio. The blue dashed line indicates 80% of circularity corresponding to an ellipse at a given aspect ratio. We empirically consider data points below the blue dashed line as strongly protrusive cells. In (B-C) 70 cells are tracked. See also *Underlying data*. ^30^

When MDA-MB-231 cancer cells are embedded in the culture insert, cells experience both the chemotactic serum gradient, and 3D contact guidance from the local fiber alignment. We set the chemical gradient by filling the inner reservoir with 20% volume concentration of serum, and filling the outer reservoir with serum free growth medium. To characterize the resulted cellular dynamics, we performed confocal live cell imaging for 18 hours after chemical gradient stabilized. On the left panel of Figure 4A we show a temporal projection of the cells where each trace represents a single cell from the beginning of recording (blue, zero hour) to the end of recording (red, 18 hours). Because the chemical and mechanical cues are in parallel, cells move in the radial direction with little excursions.

To further characterize the cell motility, we tracked the radial and tangential displacements of a random subset of cells as shown in Figure 4B. Consistent with the temporal projection, cell displacement in the tangential direction ( $\hat{\theta }$ ) is very small. The trajectories in Figure 4B also reveal that the random walk of a cell often observed in 3D ECM now occurs with reduced dimension. Cells constantly make 180 degree turns, while still showing net displacements towards the center of the device, where serum concentration is higher. Within a frame interval of one hour, cells travel at an average instantaneous velocity of 9.5 μm/hr. To quantify the efficiency of cells tracing chemoattractant gradient, we also calculated the mean chemotaxis index $\bar{\mathrm{CI}}$ :

$$\bar{\mathrm{CI}}=\hat{v}\cdot \left(-\hat{r}\right),$$ (1)

where $\hat{v}$ is a unit vector along the direction of velocity, and $\left(-\hat{r}\right)$ the direction of chemotaxis. $\bar{\mathrm{CI}}$ ranges between 1 and -1, with greater values indicating more efficient migration seeking higher chemoattractant concentration. For the cell trajectories shown in Figure 4B, we find the mean chemotaxis index equals 0.3. The tendency of moving along the serum gradient accumulates as cells navigate their microenvironment, such that over 18 hours, the average net radial displacement of cells is 85 μm towards the device center.

In addition to cell motility, we have also characterized cell morphology. In particular, we calculated the circularity and aspect ratio of binarized single cell images ( Figure 4C). Here circularity is defined as $4\pi \frac{\text{area}}{{\text{perimeter}}^{2}}$ , which equals to one for a circle and generally becomes smaller when a cell generates protrusions such as invadopodia.

We find strong ECM contact guidance coupled with a chemical cue in the parallel direction promotes cell elongation. About half of times a cell sampled in Figure 4C have aspect ratio greater than three, and in over 27% cases cells are highly elongated to have aspect ratios greater than six.

Despite of the elongation, most cells do not deviate from elliptical shape ( Figure 4C, black dashed line) to demonstrate significant surface fluctuation. This is expected as the parallel mechanochemical cues provide consistent polarizing signals for cell morphology. To better quantify the morphology of cells, we empirically classify a cell to be strongly protrusive if its circularity is less than 80% of the circularity of an ellipse with the same aspect ratio ( Figure 4C, blue dashed line). We find that 19% of cells fall into this category. Together, these results show that cell morphology can be characterized as approximately elongated ellipses.

**Figure 5.**
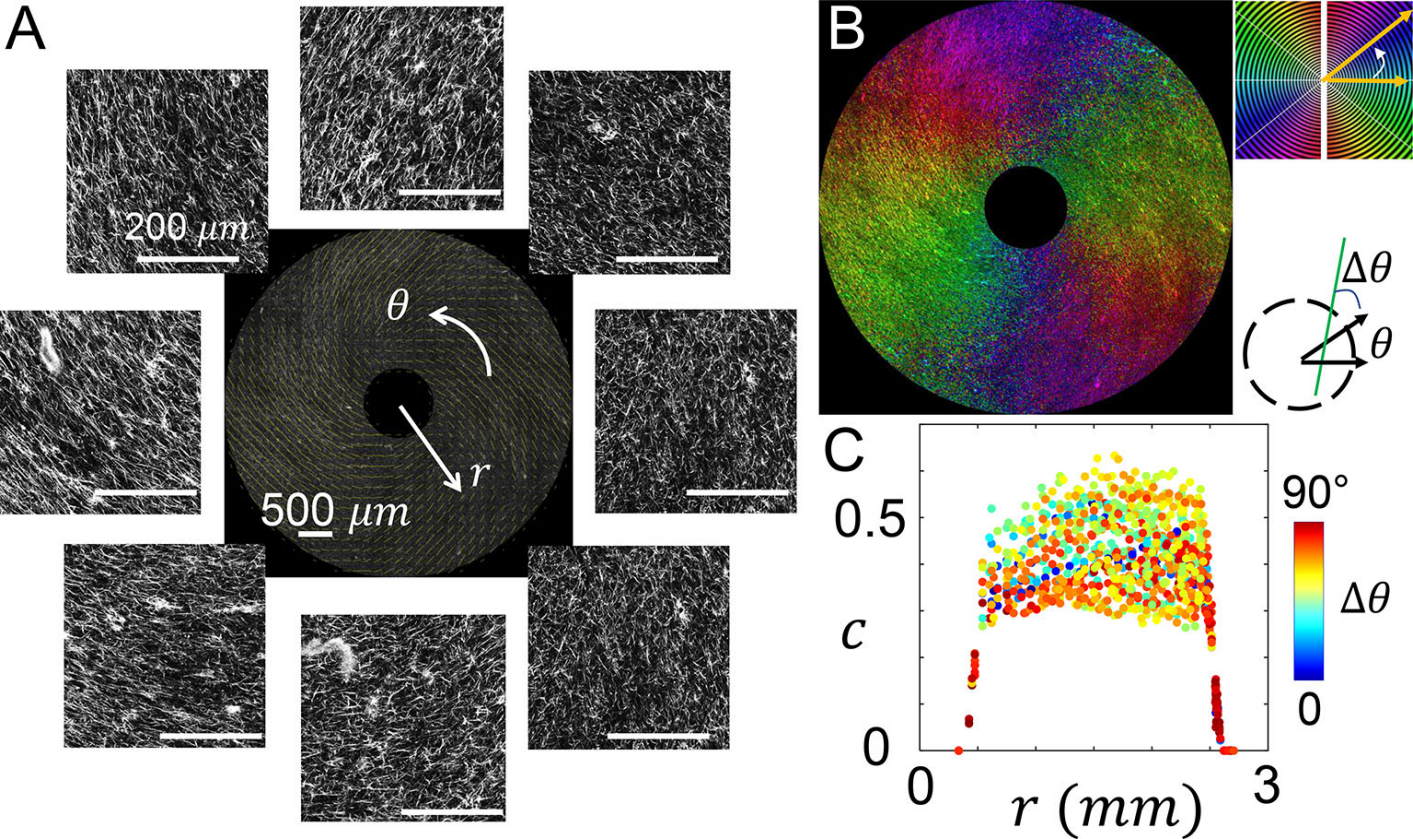
The microstructure of the vortex flow aligned extracellular matrix. (A) Confocal images showing the fluorescently labeled collagen fibers. The images are further processed to calculate the principal direction and level of alignment of collagen fibers. The center of the image is at the center of the inner reservoir, similar to Figure 3A. (B) A spatial map shows the principal direction of collagen fibers. (C) The coherence *c* at varying distances from the center of the inner reservoir. The data points are color coded by the angle Δ *θ* between local fiber direction and radial direction. See also *Underlying data*. ^31^

While the parallel chemical and mechanical cues lead to strong cell polarization and dimensional reduction of motility, we notice that in physiological condition the two cues may vary independently and form different angles. MC ^3^A allows us to conveniently investigate cellular dynamics in such microenvironment configurations. As a demonstration, we rotated the blade of MC ^3^A at a constant speed of 128 RPM, which drives the flow of collagen solution that directs the nucleation and growth of collage fibers primarily in the tangential direction. After four minutes of rotation, we lifted the blade out of the solidifying collagen solution and the ECM self-assembly was continued for the next 40 minutes. Confocal images in Figure 5A and computed local principal direction in Figure 5B show the expected fiber alignment. Note that because the global flow field resembles a vortex pattern, a radial components of fiber alignment is still observed (see also extended data Figure S7 for other rotational protocols ^32^). In most locations sampled in the device, the angle between chemical gradient (along radial direction) and contact guidance (ECM principal direction) is between 45 to 90 degrees ( Figure 5C). As a result, cells in this configuration experience chemical and mechanical cues in diverging directions. Here as in the prior case, the strength of chemical gradient was set by filling the inner reservoir with 20% volume concentration of serum, and the outer reservoir with serum free growth medium.

**Figure 6.**
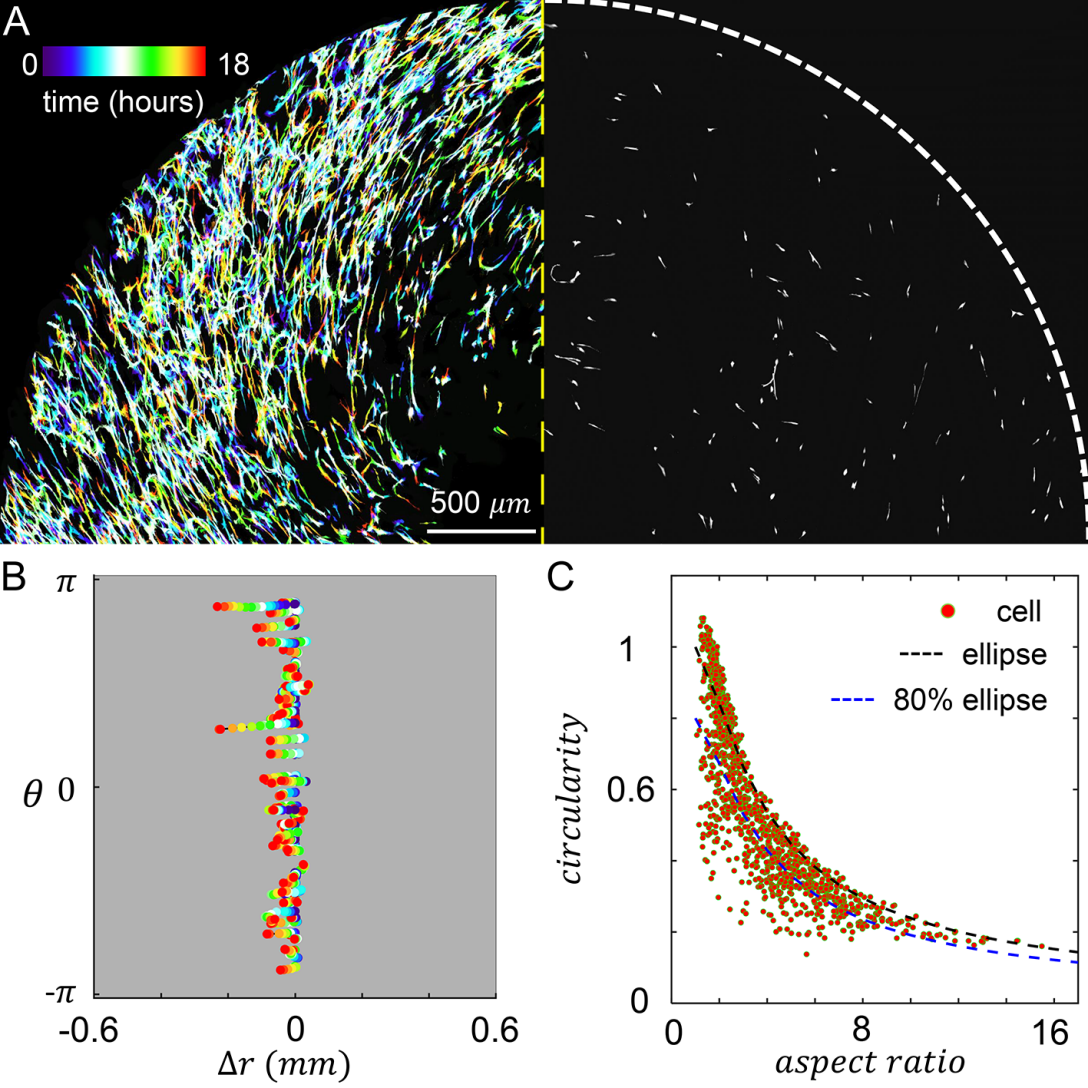
3D Cell motility is modulated by the chemoattractant gradient, which makes large angles with respect to the extracellular matrix (ECM) fiber alignment. (A) Left: temporal projected recording of MDA-MB-231 embedded in the culture insert of Modular Control of Microenvironment for Cell Migration and Culture Assay (MC ^3^A) for 18 hours. Right: a snap short at four hours. (B) Cell trajectories in the radial (Δ *r*) and azimuthal ( *θ*) coordinates. (C) A scattered plot showing cell aspect ratio and circularity. The black dashed line indicates the circularity of an ellipse at a given aspect ratio. The blue dashed line indicates 80% of circularity corresponding to an ellipse at a given aspect ratio. We empirically consider data points below the blue dashed line as strongly protrusive cells. In (B-C) 70 cells are tracked. See also *Underlying data*. ^31^

As the serum gradient drives the MDA-MB-231 cells radially inward, ECM fibers present contact guidance that steers the cells in the tangential direction. The temporal projected confocal recording demonstrates the joined effects of mechanical and chemical cues to cell motility ( Figure 6A). We track a random subset of 70 cells as shown in Figure 6B. Compared with the previous configuration where collagen fibers align radially, cells in the current configuration exhibit pronounced migration that vary their azimuthal angles. Indeed, instantaneous velocity (approximated by the displacement between one hour frame intervals) shows a mean chemotaxis index of 0.14, less than half of the value for radially aligned ECM. Over the course of 18 hours, the net radial displacement averaged over all tracked cells is 35 μm towards the device center, which is again less than half of the value for radially aligned ECM.

The diverging mechanochemical cues also modulate the cell morphology. In particular, only 17% cells sampled have aspect ratios greater than six, compared with 27% in the presence of parallel mechanochemical cues. Additionally, of all the cells sampled 26% show characteristics of strongly protrusive cells, compared with 19% in the case of radially aligned ECM. Together, these results show that when contact guidance and chemotaxis are along diverging directions, cells show significant reduction in their migration along the chemical gradient. At the same time, cells tend to demonstrate small aspect ratio shapes while featuring strong surface fluctuations.

## Conclusions

Many biological processes involve cells to navigate 3D ECM, which contains multiplexed environmental cues. ^22^^–^^24^ It is conceivable that modeling the cross-talk of biochemical and biophysical factors will improve the physiological relevance of *in vitro* cell-based assays. Here we present a modular platform that allows one to conveniently pattern the microstructure of 3D ECM, so that contact guidance from the ECM fiber alignment and spatial gradient of soluble factors can be independently controlled to jointly modulate the cellular dynamics.

Our platform, MC ^3^A, generates sustained chemical gradient over more than 18 hours under passive diffusion. MC ^3^A features an open channel design, which makes it easy to deliver soluble factors to the cells, and to extract samples for downstream analysis such as sequencing. This is in contrast to other microfluidics culture and chemotaxis platforms, ^25^ where the cells in the closed channels are often difficult to be retrieved, especially when they are embedded in solidified matrices. Additionally, the reservoirs are easily accessible so that additional solutions can be brought in externally to generate time-dependent chemical environment or to simply extend the duration of stable gradient.

In MC ^3^A, cells are cultured in thick layer of ECM (>500 μm) that provide true 3D support. This is in contrast to many microfluidics-based chemotaxis device where the limited channel depth, often less than 100 μm, can not sufficiently screen the mechanical effects of rigid boundaries. ^26^^,^^27^

In MC ^3^A we optimize the geometric design of the boundaries of both spinning blade and dish insert such that the ECM microstructure can be easily controlled through the programmable rotational protocols of the blade. This approach avoids pre-loaded mechanical stress in the matrix when fiber alignment are induced by external stress. ^28^ Compared with other flow-based ECM aligning methods, such as magnetomicrofluidics, ^17^ MC ^3^A produce uniform ECM alignment over a much larger spatial range (three times more effective area than in, ^22^ and five times more area than in ^22^). MC ^3^A is also compatible with most tissue-derived proteins (such as collagen, matrigel and fibronectin) or synthetic hydrogels. Therefore the user can take full advantage of current and future progress in tissue-mimicking biomaterials.

MC ^3^A utilizes a modular design such that the shape of the blade, and the geometry of the culture insert can be altered for expanded functionalities. As an example, we have constructed a device to fit two separate sets of ECM with their inner reservoirs connected (see also extended data Figure S6 ^32^). With different combinations of medium in each reservoir, we can use this dish insert to run replicating experiments or to make side-by-side comparison between distinct microenvironments. Because we make dish inserts through standard 3D printing, MC ^3^A allows rapid prototyping to explore expanded functionality.

In summary, MC ^3^A provides a simple and reliable platform to program complex 3D tissue-mimicking microenvironment. Given the importance of multiplexed chemical and mechanical cues, we think MC ^3^A will advance the state-of-the-art of *in vitro* models in drug screening, regenerative medicine, and many areas of fundamental research.

## Data availability

### Underlying data

• Figshare: Chemical gradient. https://doi.org/10.6084/m9.figshare.21049981 ^29^
  This project contains the following underlying data:
‐ chemical gradient.zip. This file contains raw microscopy images of showing the diffusion of a tracer dye (fluorescein) in the collagen ECM of a MC ^3^A device.
• Figshare: Radially aligned sample. https://doi.org/10.6084/m9.figshare.20112950 ^30^
  This project contains the following underlying data:
‐ ECM microstructure snapshot.zip. This file contains raw microscopy images of collagen ECM aligned radially in a MC ^3^A device.
‐ Cell 3D cultured snapshot.zip. This file contains raw microscopy images of MDA-MB-231 cells cultured in collagen ECM that is aligned radially in a MC ^3^A device.
‐ Cell migration timelapse.zip. This file contains time lapse recording images of MDA-MB-231 cells migrating in collagen ECM that is aligned radially in a MC ^3^A device.
• Figshare: Tangentially aligned sample. https://doi.org/10.6084/m9.figshare.20112989 ^31^
  This project contains the following underlying data:
‐ ECM microstructure snapshot.zip. This file contains raw microscopy images of collagen ECM aligned tangentially in a MC ^3^A device.
‐ Cell 3D cultured snapshot.zip. This file contains raw microscopy images of MDA-MB-231 cells cultured in collagen ECM that is aligned tangentially in a MC ^3^A device.
‐ Cell migration timelapse.zip. This file contains time lapse recording images of MDA-MB-231 cells migrating in collagen ECM that is aligned tangentially in a MC ^3^A device.

### Extended data

• Figshare: Supplementary Materials. https://doi.org/10.6084/m9.figshare.20915452 ^32^
  This project contains the following underlying data:
‐ SI_final.pdf. This file includes additional information regarding the device manufacture and additional experimental details.

Data are available under the terms of the Creative Commons Attribution 4.0 International license (CC-BY 4.0).
