# Supplementary material for: Patterning ECM microstructure to investigate 3D cellular dynamics under multiplexed mechanochemical guidance
Source: F1000Res. 2022 Sep 20;11:1071. [Version 1] doi: 10.12688/f1000research.125171.1 (PMC10603315; doi:10.12688/f1000research.125171.1)
Supplement: Peer review history

# Reviewer report: Reviewer response for version 1

**DOI:** 10.5256/f1000research.137449.r206910

**Published:** 2023-10-26

**Authors:** Suzanne M Ponik

**Affiliations:** 1 University of Wisconsin-Madison, Madison, Wisconsin, USA

The manuscript entitled, “Patterning ECM microstructure to investigate 3D cellular dynamics under multiplexed mechanochemical guidance”, outlines the development of a platform for 3D cell culture that can modulate chemical and mechanical cues respectively. This novel device can be used to study the relative contribution of chemotaxis and contact guidance on cell morphology and migration.  The ability to independently control fiber alignment vs chemotaxis provides opportunities to address unanswered questions about how these cues impact cell morphology and migration. The utility of the platform is of interest to researchers in this field; however, the manuscript lacks detail in the description of methods and lacks experimental evidence of reproducibility. Addressing these shortcomings is required prior to publication.

 1. For a manuscript that defines the development of a novel platform the methodology is extremely vague.   

- As written, it is very unclear how radial vs tangential fiber architecture is achieved.  Some aspects of the mechanical perturbations (rotation of the blade) are provided in the methods section, while some are in the results text. The descriptions of each condition seem to overlap.  Please define both radial and tangential fiber alignment protocols in the methods section. (blade rotation speed, duration of spinning, whether collagen is unpolymerized at the time of spinning, the polymerization conditions, and the exact timing of serum addition vs initiation of image acquisition)

 

- Collagen labeling methods are not clear.  The FITC-labeled collagen from AnaSpec is a quenched collagen, which fluoresces upon enzymatic cleavage (MMP activity).  Presumably diluting this collagen into high-concentration Collagen Type I alleviates the quenched fluorescence so that a subset of fibers are fluorescent.  These details should be added to the methods section.

 

- The cell concentration is not defined.  “…we suspend cells at low density in neutralized collagen solutions.”  For reproducibility, a cell concentration should be defined.

2.  It is not clear that the authors have reproduced the alignment conditions.  The experimental N is not reported.  How reproducible is the fiber alignment? Chemical gradients?  The tangential alignment appears quite variable (45-90 degrees), which is a large margin. The analysis of chemical gradients and alignment should be repeated in at least 3 experiments.  Furthermore, were all cell metrics acquired from one device for tangential and one device for radial alignment? Similarly, the migration speeds/distances for radial vs tangential fiber alignment should be repeated across a minimum of 3 independent experiments.    

 3. The authors state that cellular protrusions are altered between alignment/chemical gradient conditions.  However, images were collected with a 10x objective every hour for 18hrs. Protrusion dynamics in 3D have been reported to occur on the scale of minutes.  Higher magnification and shorter imaging intervals are necessary to ensure that cellular protrusion dynamics are accurately quantified.

 4. Finally, if the method is interpreted correctly by this reviewer, the cells are subjected to blade rotation for either 0 or 4 minutes.  The authors do not account for the application of direct mechanical stress on the cells in their response to tangential alignment. This is an important factor that needs to be controlled or minimally discussed.

Is the work clearly and accurately presented and does it cite the current literature?

Partly

If applicable, is the statistical analysis and its interpretation appropriate?

Not applicable

Are all the source data underlying the results available to ensure full reproducibility?

Yes

Is the study design appropriate and is the work technically sound?

No

Are the conclusions drawn adequately supported by the results?

Partly

Are sufficient details of methods and analysis provided to allow replication by others?

No

Reviewer Expertise:

Mechanotransduction, 3D collagen cell culture, image analysis, cell migration and invasion, cancer cell biology.

I confirm that I have read this submission and believe that I have an appropriate level of expertise to confirm that it is of an acceptable scientific standard, however I have significant reservations, as outlined above.

# Reviewer report: Reviewer response for version 1

**DOI:** 10.5256/f1000research.137449.r206911

**Published:** 2023-10-26

**Authors:** Nicholas Kurniawan

**Affiliations:** 1 Eindhoven University of Technology, Eindhoven, The Netherlands

In this manuscript, Esfahani and Sun described a method to create an experimental platform where cell response to gradient in chemical factors and to the organization of extracellular environment can be decoupled. This is interesting as cells are known to sense and respond to both factors, but the interplay between them is difficult to study systematically. Thus, the approach and the insights obtained from it can be very useful to the community. The work and analyses are solid and were performed nicely, and the manuscript is a pleasure to read. My only suggestions are to include some pertinent missing information, which are crucial for the readers to understand the study.

1. The working mechanism of the device and how it can be used to tune collagen microstructure can be better explained. It is not clear how the blade and boundary condition of the device alone (apparently even without rotation) can already create fiber alignment.
2. The authors state that the value of coherence c is highly affected by noise in the image and therefore can only reach a max value of 0.2 rather than the theoretical 1.0. Some examples of images with different levels of noise and the corresponding c value will be nice (perhaps as a supplementary figure).
3. The authors stated that the tip shape of the blade was optimized to guide the collagen gelation. Since the blade shape appears to be critical to the whole mechanism of the approach, description of the different tip shapes that were tried and the final tip shape and dimensions used will be very useful.
4. It will be great if dimensions are added to the schematic in Figure 1.
5. Since the goal of the study is to study the individual and combined effects of chemotaxis and collagen orientation, it will be most interesting to compare the anisotropic (Figure 3,4) vs. isotropic condition (not included yet) and chemotaxis (Figure 5,6) vs. no chemotaxis (not included yet). Basically including the outcomes of the control conditions.
6. The chemical gradient experiment was done using rhodamine B as a proxy for diffusive factor’s concentration profile. It will be informative to mention the diffusivity and molecular size of rhodamine B as compared to those of chemoattractants normally present in the context of cancer.
7. The chemical gradient experiment was performed only for one condition (isotropic gel), which is different from the conditions used in the rest of the paper (anisotropic gel). It will be more convincing to include results from the anisotropic gels too (those in Figure 3 and in Figure 5).
8. Information about how the chemical gradient experiment was performed is missing in Methods. Also, at what height were the images in Figure 2 taken to analyze the fluorescence intensity?
9. Regarding the experiments described in Figure 4 and 6, as far as I understand the chemical gradient is positive (serum is at the center of the device). So why did the cells move outwards (away from serum) and not inwards (towards serum)? What is the zero position in Figure 4B and 6B?
10. Serum has been previously reported to affect collagen gelation, resulting in different collagen fiber thickness and microstructure. In the presence of serum concentration gradient, could it be that there is also a gradient in collagen fiber formation and microstructure, which could potentially also affect cancer cell migration? If the authors have characterized the collagen at different radial positions, this will be nice to add. Otherwise, at least the authors could comment on this.
11. I think it will be more precise if the authors indicate that the approach specifically allows radial-circumferential patterning of collagen and radial chemical gradient (and not some other axis).

Is the work clearly and accurately presented and does it cite the current literature?

Yes

If applicable, is the statistical analysis and its interpretation appropriate?

Yes

Are all the source data underlying the results available to ensure full reproducibility?

Yes

Is the study design appropriate and is the work technically sound?

Partly

Are the conclusions drawn adequately supported by the results?

Partly

Are sufficient details of methods and analysis provided to allow replication by others?

Partly

Reviewer Expertise:

biomedical engineering

I confirm that I have read this submission and believe that I have an appropriate level of expertise to confirm that it is of an acceptable scientific standard, however I have significant reservations, as outlined above.

# Reviewer report: Reviewer response for version 1

**DOI:** 10.5256/f1000research.137449.r151070

**Published:** 2022-11-04

**Authors:** Jiandi Wan

**Affiliations:** 1 Department of Chemical Engineering, University of California, Davis, CA, USA

The authors described a novel 3D cell culture system and characterized its application to control independently ECM fiber alignment and spatial gradient of soluble factors, which is an important contribution to the field. The manuscript is well written and clearly demonstrates the advantages of the new platform.

 One minor comment: it will be helpful to show representative images of cell morphology when there is a diverge between the directions of ECM alignment and serum gradient.

Is the work clearly and accurately presented and does it cite the current literature?

Yes

If applicable, is the statistical analysis and its interpretation appropriate?

Not applicable

Are all the source data underlying the results available to ensure full reproducibility?

Yes

Is the study design appropriate and is the work technically sound?

Yes

Are the conclusions drawn adequately supported by the results?

Yes

Are sufficient details of methods and analysis provided to allow replication by others?

Yes

Reviewer Expertise:

Microfluidic

I confirm that I have read this submission and believe that I have an appropriate level of expertise to confirm that it is of an acceptable scientific standard.
